# Nonthermal plasma treated solution inhibits adipocyte differentiation and lipogenesis in 3T3-L1 preadipocytes via ER stress signal suppression

**DOI:** 10.1038/s41598-018-20768-5

**Published:** 2018-02-02

**Authors:** Sung Un Kang, Haeng Jun Kim, Dae Ho Kim, Chang Hak Han, Yun Sang Lee, Chul-Ho Kim

**Affiliations:** 1 Department of Otolaryngology, School of Medicine, Ajou University, Suwon, Republic of Korea; 2 Department of Molecular Science & Technology, Ajou University, Suwon, Republic of Korea

## Abstract

The accumulation and differentiation of adipocytes contribute to the development of obesity and metabolic diseases. It is well-known that interactions of transcription factors such as peroxisome proliferator-activated receptor gamma (PPARγ), CCAAT/enhancer binding protein α (C/EBPα), and endoplasmic reticulum (ER) stress are required for adipogenesis. Recently, use of nonthermal atmospheric plasma (NTP) is expanding from the biomedical field into various other fields. In this study, we investigated whether nonthermal plasma-treated solution (NTS) has an inhibitory effect on adipogenesis and elucidated its mechanisms. Our results demonstrated that NTS significantly inhibited pre-adipocyte differentiation into adipocytes based on Oil Red O staining and triglyceride accumulation. Moreover, NTS treatment suppressed the mRNA and protein expression levels of key adipogenic transcription factors, and adipocyte-specific genes. NTS also down-regulated endoplasmic reticulum stress-related proteins. Consistent with *in vitro* studies, an animal study using a mouse model of diet-induced obesity showed that NTS treatment reduced body weight and fat, ER stress/UPR, triglyceride, and adipogenic marker level without altering food intake. These findings indicate that NTS inhibits adipogenic differentiation, and provide a mechanistic explanation of the inhibitory effect of NTS on adipogenesis. Taken together, our results suggest that NTS might be useful to treat obesity and obesity-related diseases.

## Introduction

Obesity is one of the most common metabolic disorders worldwide; it has been suggested that obesity is directly related to increased prevalence of various adult diseases such as type 2 diabetes, hypertension, hyperlipidemia, heart disease, and cancer^1^. These diseases are called metabolic syndrome or insulin resistance syndrome; they are caused by atherosclerosis or cardiovascular disease. Obesity is a metabolic disease that occurs due to unbalanced intake and consumption of calories. It is characterized by increased adipose cell size (hypertrophy) or number (hyperplasia)^2^. The most well-known mechanism of obesity is that adipocytes differentiate into pre-adipocytes, and when storage is needed, adipocytes increase in number through proliferation and differentiation from pre-adipocytes. Adipocyte differentiation has been studied using cells such as 3T3-L1 mouse pre-adipocytes. CCAAT/enhancer binding protein α (C/EBPα) and peroxisome proliferator-activated receptor γ (PPARγ) are two well-known adipogenic transcription factors that regulate adipogenic differentiation^3–5^. The initial event in adipogenesis is driven by adipogenic differentiation of C/EBPβ and C/EBPδ in response to adipogenic stimuli in MDI(3-isobutyl-1-methylxanthine, dexamethasone, and insulin). C/EBP transcription factors then stimulate the expression of PPARγ and C/EBPα, which cross-regulate each other to induce adipocyte-specific expression of genes such as FAT, FAS, and ACC^6,7^. This gene expression then stimulates the formation of mature adipocytes with triglyceride accumulation and lipid droplet formation.

Many studies have focused on how to inhibit adipocyte proliferation and differentiation. In particular, inhibiting transcription factors and adipokines that mediate or modulate adipogenesis is one of the best ways to develop new therapeutic drugs for obesity. Recently, some studies have suggested that endoplasmic reticulum (ER) stress and unfolding protein response (UPR) also play an important role in adipogenic differentiation^6–8^.

ER is an intracellular organelle in which fat, carbohydrate, cholesterol, and protein are regulated by various signals related to metabolism. Protein synthesis also occurs in ER. Mechanisms such as molecular chaperones are involved in synthesizing precisely structured proteins. The ER is also a site where triglyceride droplets are formed^9^. Thus, cells with ER protein expression are more advanced than cells that do not^10^.

In the process of differentiation from pre-adipocytes into adipocytes, the role of the ER is important because more peptides and lipid mediators associated with differentiation are needed.

It has been suggested that during adipogenesis ER stress markers such as protein kinase RNA-like endoplasmic reticulum kinase (PERK), inositol requiring enzyme 1 (IRE1), and activating transcription factor 6 (ATF6) are upregulated in adipocytes and adipose tissue^11–13^.

A recent study showed that the PERK pathway is required for adipocyte differentiation. Activated PERK phosphorylates Ser51 of eIF2α to reduce protein synthesis. It also increases mRNA translational of ATF4 and induces CHOP transcription^14^.

Accumulating evidence has revealed PERK-dependent regulation of lipogenesis during mouse mammary gland development and adipocyte differentiation^14^. PERK is overexpressed in the mouse pancreas; PERK-deficient mice have shown neonatal diabetes mellitus that caused exocrine pancreatic atrophy^15^. Interestingly, a chemical chaperone and ER stress inhibitor can affect adipocyte differentiation^6^. Adipocytes are suppressed by injecting the molecular chaperone that can suppress ER stress in DB/DB mice.

In addition, several studies have reported that ER stress markers such as XBP1 are upregulated by ATF6 and IRE1 signaling to regulate lipogenesis in the liver^16^. However, how ER stress and UPR activation influence adipogenesis is not fully understood yet.

Plasma is an ionized gas composed of charged particles, electron excited atoms, molecules, radicals, and UV photons. It is known as the fourth state of matter after liquid, solid, and gas^17^. A recent study showed that plasma can be used for biomedical applications, and NTP specifically, has been studied for clinical application in recent decades. In our previous study, we demonstrated that NTP could control apoptosis and tumor environment of cancer cells^18,19^. In addition, it has been reported that NTP induced wound healing and muscle cell differentiation^20^. However, the effect of plasma on adipocyte differentiation has not been reported yet.

The involvement of ER stress and UPR in adipogenesis has been reported in some studies^15,16,18^. However, to the best of our knowledge, their potential to inhibit adipogenesis using plasma treatment has not been reported yet.

Therefore, the objective of this study was to determine whether NTS could inhibit adipogenic differentiation *in vitro*, and *in vivo* and elucidate how it might influence ER stress inhibition and UPR activation signaling.

## Materials and Methods

### Cells line and Reagents

3T3-L1 pre-adipocytes were purchased from the American Type Culture Collection (Manassas, VA, USA). 3T3-L1 cells were maintained in Dulbecco’s Modified Eagle’s Medium (GIBCO, Carlsbad, CA, USA) supplemented with 10% calf serum and penicillin-streptomycin (100 U/ml, GIBCO) at 37 °C with 5% CO_2_ under humidified conditions. Upon confluence, the growth medium was changed the following day and replaced with differentiation medium consisting of 0.5 mM 3-isobutly-1-methylxanthine (IBMX, Sigma Aldrich, St. Louis, MO, USA), 1 mM dexamethasone, 10% fetal bovine serum (FBS, GIBCO), and insulin (10 mg/ml, Sigma Aldrich). The medium was changed every three days thereafter.

### Design of nonthermal plasma treated solution

**Figure 1:**
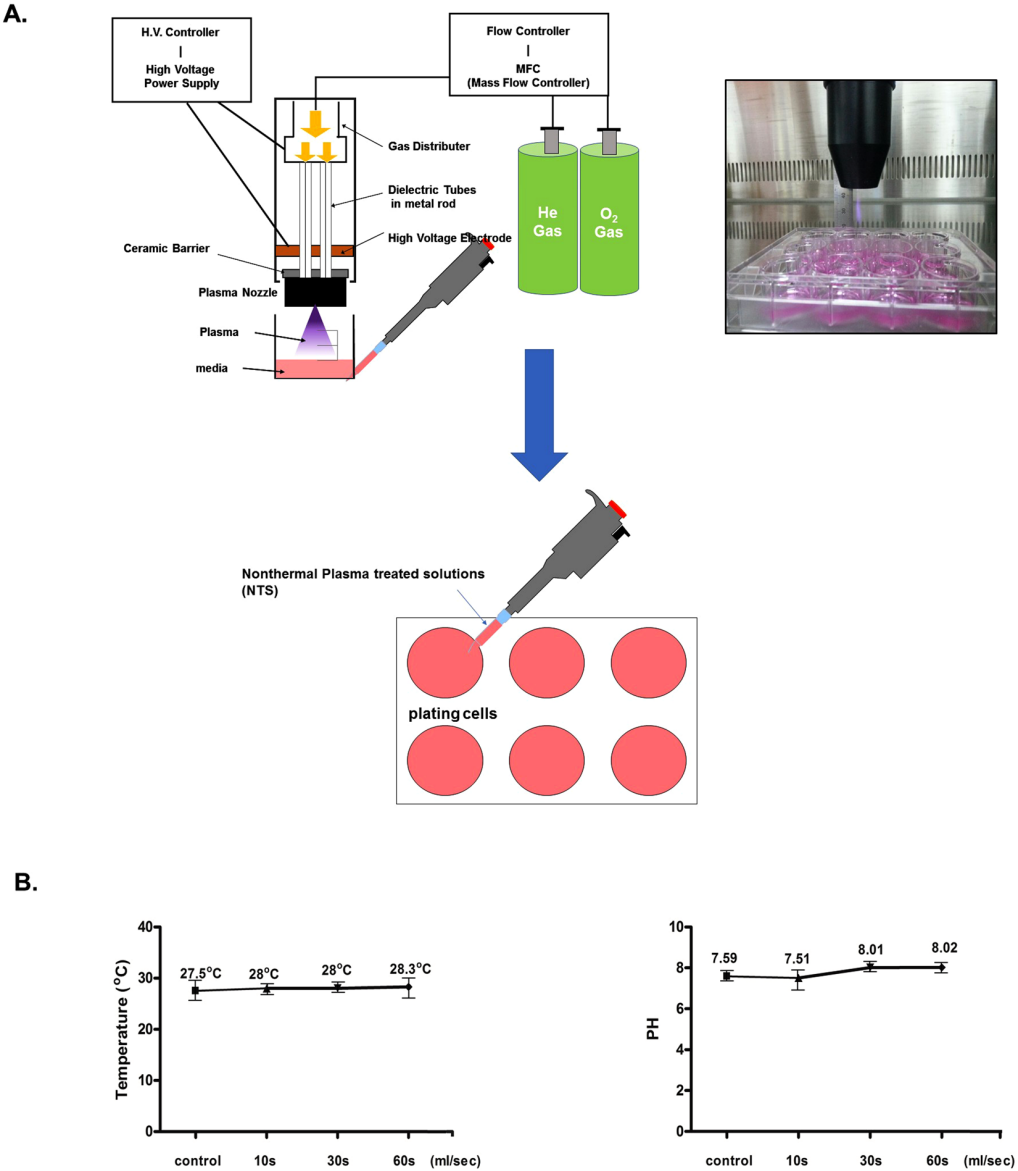
(**A**) Diagram of the developed He and O_2_-based NTS system and scheme as well as photo depicting the experimental design and preparation of NTS. (**B**) Temperature and pH changes at various time points (10 s, 30 s, and 60 s) measured with a non-contact IR thermometer and pH meter.

In this study, we designed a nonthermal plasma treated solution (NTS) system based on our previous study of its biological research applications (Fig. 1A). We used helium and oxygen as carrier gases^17^.

The plasma device was equipped with a pair of electrodes made of Al_2_O_3_ (high-voltage and ground electrodes, 10 × 40 mm^2^ dimension, 2 mm gap between electrodes) that was isolated from direct contact with the plasma by a ceramic barrier.

We used helium and oxygen as carrier gases for the plasma treated solution because we previously found that adding it could improve cell permeation efficiency inside the medium^21^. For NTS treatment, we added 10 ml of cell medium to a petri dish (100 mm diameter, TPP, Renner, Dannstadt, Germany). The distance between the plasma device and the bottom of the petri dish was about 4 cm.

NTP treatment time was 1 minute per ml. Specifications of the power supply with this system were: 2 kV minimum, 13 kV maximum, and mean frequency 20–30 kHz. For this study, we used 4 kV power.

Temperature and pH changes of NTS are shown in Fig. 1B. The temperature of the medium in which we generated the NTS started at 27.5 °C; after one minute, we changed it to 28.3 °C. The pH started at 7.59, and we measured it to be 8.02 after one minute.

### Lipid accumulation assay

We measured fat accumulation by Oil Red O staining (Sigma Aldrich, St. Louis, MO, USA) and found differentiated cells in the NTS in various concentrations. We washed the cells with phosphate-buffered saline (PBS) fixed with 10% formalin and stained them with 60% Oil Red O for 20 min at room temperature. We then washed the stained cells with distilled water and photographed them using the EVOS FL auto cell imaging system (Thermo Fisher Scientific, Waltham, MA, USA). We dissolved Oil Red O dyes in 100% isopropanol. We measured absorbance at a wavelength of 520 nm using an ELISA reader (Bio–Tek, Winooski, VT, USA). Fat accumulation measurement rate was expressed as a percentage of the control.

### Triglyceride contents assay

We measured triglyceride content using a triglyceride colorimetric assay kit (Cayman, Ann Arbor, MI, USA). We washed the cells three times with 1% Triton X-100 in PBS, pH 7.4. After we homogenized the cell suspension by sonication for 5 min, we assayed the cell lysate to determine triglyceride content, which we measured following the manufacturer’s instructions.

### Immunofluorescence assay

We cultured 3T3-L1 cells on cover slips (Thermo Fisher Scientific, Rochester, NY, USA), differentiated them, and treated them with NTS (1 min/ml) or vehicle control. At 24 h after incubation, we fixed the cells with 4% formaldehyde and blocked with 5% bovine serum albumin (Millipore, Bedford, MA, USA) in PBS for 1 h. We then incubated the cover slips with polyclonal rabbit anti-PPARγ or perilipin antibody (1:100, Cell Signaling, Danvers, MA, USA) for 2 h, washed them with PBS, and incubated them with Alexa 546 and Alexa 488-labeded antibody (1:500, Molecular Probe, Eugene, Oregon, CA, USA) for 1 h. After we rinsed the slips in PBS, we added a Hoechst 33258 Molecular Probe to them and incubated at room temperature for 15 min to counterstain the nuclei. We washed the slides with PBS, mounted them with Vectashield (Vector Laboratories, Inc., Burlingame, CA, USA), and visualized them using a fluorescence microscope (EVOS FL Auto, Thermo Fisher).

### Western blot

We lysed the cells in RIPA buffer (Sigma Aldrich) containing 150 mM NaCl, 1.0% Nonidet-P 40, 0.5% sodium deoxycholate, 0.1% sodium dodecyl sulfate, 50 mM Tri (pH 8.0), protease inhibitor cocktail, and PhoSTOP (Roche Molecular Biochemicals, Basel, Switzerland) as described previously^17^. We used the following primary antibodies: PPARγ, C/EBPα, CHOP, GRP78, PERK, p-PERK, p-eIF2α, eIF2α, IRE1α, IRE1α, FABP4, perilipin, and α-tubulin (1:1000; Cell Signaling Technology, Danvers, MA, USA).

Secondary antibodies (anti-rabbit IgG or anti-mouse IgG, 1:2000)) were purchased from Cell Signaling Technology. We conducted immunoreactive detection of specific proteins using an ECL Western blotting kit (GE, Hercules, CA, USA) according to the manufacturer’s instructions.

### Quantitative real-time PCR analysis

We extracted total RNA from 3T3-L1 cells using TRIzol®reagent (Gibco-BRL, Grand Island, NY, USA). We mixed total RNA (1 μg) with 10 μl of ReverTrace qPCR RT (Toyobo Co. Ltd., Osaka, Japan) mixture for cDNA synthesis according to the manufacturer’s instruction. We quantified the targeted genes with one-step real-time PCR using a Lightcycler 96 (Roche Molecular Biochemicals, Basel, Switzerland). We used the following specific primers: PPARγ Forward, 5′-TTCAGCTCTGGGATGACCTT-3′; PPARγ Reverse, 5′- CGAAGTTGGTGGGCCAGAAT-3′, C/EBPα Forward, 5′- GTGTGCACGTCTATGCTAAACCA-3′; C/EBPα Reverse 5′-GTTAGTGAAGAGTCTCAGTTTG-3′, Acetyl-CoA carboxylase Forward 5′ GCGTCGGGTAGATCCAGTT-3′; Acetyl-CoA carboxylase Reverse 5′- CTCAGTGGGGCTTAGCTCTG-3′, fatty acid synthase Forward 5′- TTGCTGGCACTACAGAATGC-3′; fatty acid synthase Reverse 5′- AACAGCCTCAGAGCGACAAT-3′, FAT Forward, 5′-TAGTAGAACCGGGCCACGTA-3′; FAT Reverse, 5′-CAGTTCCGATCACAGCCCAT-3′, SCD1 Forward, 5′- CATCGCCTGCTCTACCCTTT-3′; SCD1 Reverse, 5′-GAACTGCGCTTGGAAACCTG–3′.

### Cell cytotoxicity analysis

To investigate NTS cytotoxicity, we used MTT (3-(4,5-dimethylthiazol-2-yl)-2,5-diphenyl-tetrazolium bromide, Sigma-Aldrich) as described previously^17^. Briefly, we seeded 3T3-L1 pre-adipocytes into 96-well cell culture plates. After we induced differentiation, we treated the cells with NTS or vehicle. Cell viability results were presented as percentages normalized to untreated cells.

### Annexin V–fluoresceinisothiocyanate/propidiumiodide detection of apoptotic cells

Apoptotic cells were detected using an AnnexinV–fluoresceinisothiocyanate/propidiumiodide (PI apoptosis) detection kit (BD Biosciences, Bedford, MA, USA). Briefly, we plated cells in 6-well culture dishes and treated them with NTS or vehicle followed by incubation for 24 h. We then prepared samples according to the manufacturer’s protocol. We detected apoptosis using a BD FACS Aria III instrument (BD Biosciences) with excitation and emission wavelengths at 488 and 530 nm, respectively.

### TUNEL assay

We treated cells grown on cover slips with NTS or vehicle for 24 h, fixed them in 4% paraformaldehyde at room temperature for 1 h, and subjected them to DNA fragmentation analysis using an *in situ* cell death detection kit (Roche Molecular Biochemicals) according to the manufacturer’s instructions. We visualized stained cells with a fluorescence microscope (CarlZeiss, Oberkochen, Germany). Then we randomly selected digital images of apoptotic cells.

### *In vivo* studies

Male five-week-old C57BL/6 mice were purchased from Orient Bio Co. Ltd (SungNam, Korea). The animals were randomly assigned to a normal diet (ND approximately 10% energy as fat, *N* = 10) or a high-fat diet (HFD approximately 60% energy as fat, N = 10) for 5 weeks. Each group was divided into two groups, one with PBS (*N* = 10) and the other with NTS (N = 10). The animals were treated with 200 μl of NTS everyday by intraperitoneal injection. Body weight and feed consumption were measured every other day. Epididymal white adipose tissue (eWAT) and inguinal white adipose tissue(ingWAT) were obtained from the mice at indicated times for further analysis. For histology and the measurement of adipocyte size, 5–10 representative images per slide were captured and at least 500 cells were analyzed using the Image J software (area in pixels). Animal care and procedures were in accordance with the National Institutes of Health Guidelines for the Care and Use of Laboratory Animals, and all experiments were approved by the Committee for Ethics in Animal Experiments of the Ajou University School of Medicine (2017-0025).

### Statistical analysis

We performed one-way analysis of variance (ANOVA) following post hoc Tukey’s test using SPSS 20.0 statistical software (SPSS, Chicago, IL, USA). Data parameters from three independent experiments are expressed as the mean ± S.D. We considered *P* < 0.05 statistically significant (*P < 0.05; **P < 0.01; ***P < 0.001).

## Results

### NTS has no significant cytotoxicity to 3T3-L1 cells

**Figure 2:**
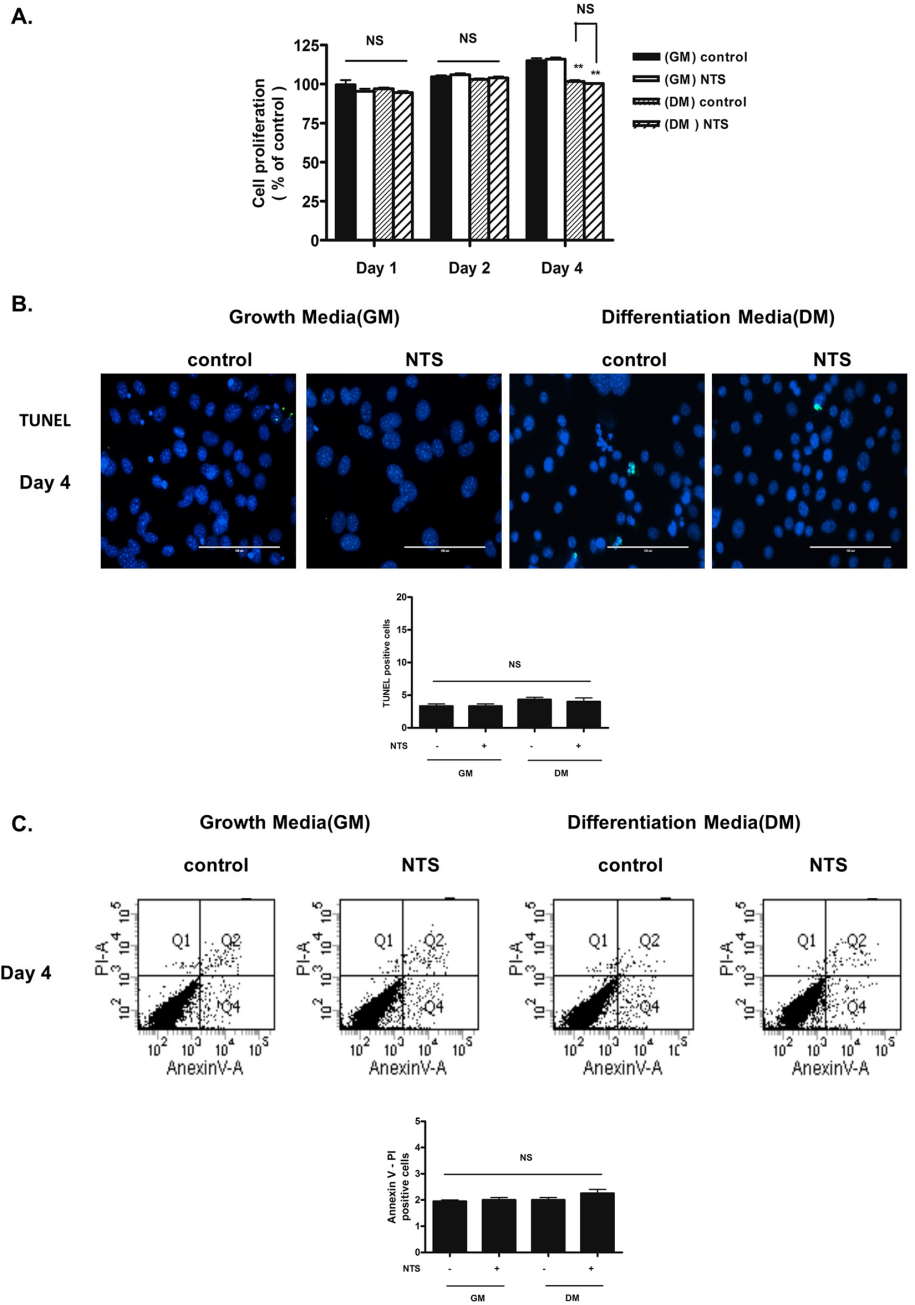
Evaluation of cell cytotoxicity and apoptotic effects of NTS (4 kV for 1 ml/1 min) on growth media (GM) and differentiation media (DM) in 3T3-L1 pre-adipocyte cells. (**A**) Cell proliferation and cytotoxicity were determined by MTT assay after NTP treatment. Bar graph presents mean ± standard deviation of three independent experiments. NS, not significant; **P < 0.01. (**B**,**C**) 3T3-L1 pre-adipocytes were treated with NTS on GM and DM three times and further incubated for 96 h from the initial exposure. Apoptosis (**B**) was determined by TUNEL assay. Scale bars = 100 μm. (**C**) Annexin V/PI assay and pre-adipocyte quantification. Bar graph: mean ± S.D. of three independent experiments. NS, not significant.

First, we determined whether NTS had a cytotoxic effect on adipocytes. Apoptotic cell death analysis showed that NTS had no cytotoxicity to 3T3-L1 cells (Fig. 2A, Supplementary Fig. 1).

In order to confirm that NTS had no cytotoxicity, we performed staining with Annexin V-PI (an early apoptotic marker) and TUNEL assay (markers of late apoptotic cells). Results showed that NTS treatment did not induce apoptotic 3T3-L1 cell death (Fig. 2B,C).

### NTS inhibits adipogenic differentiation of 3T3-L1 cells

**Figure 3:**
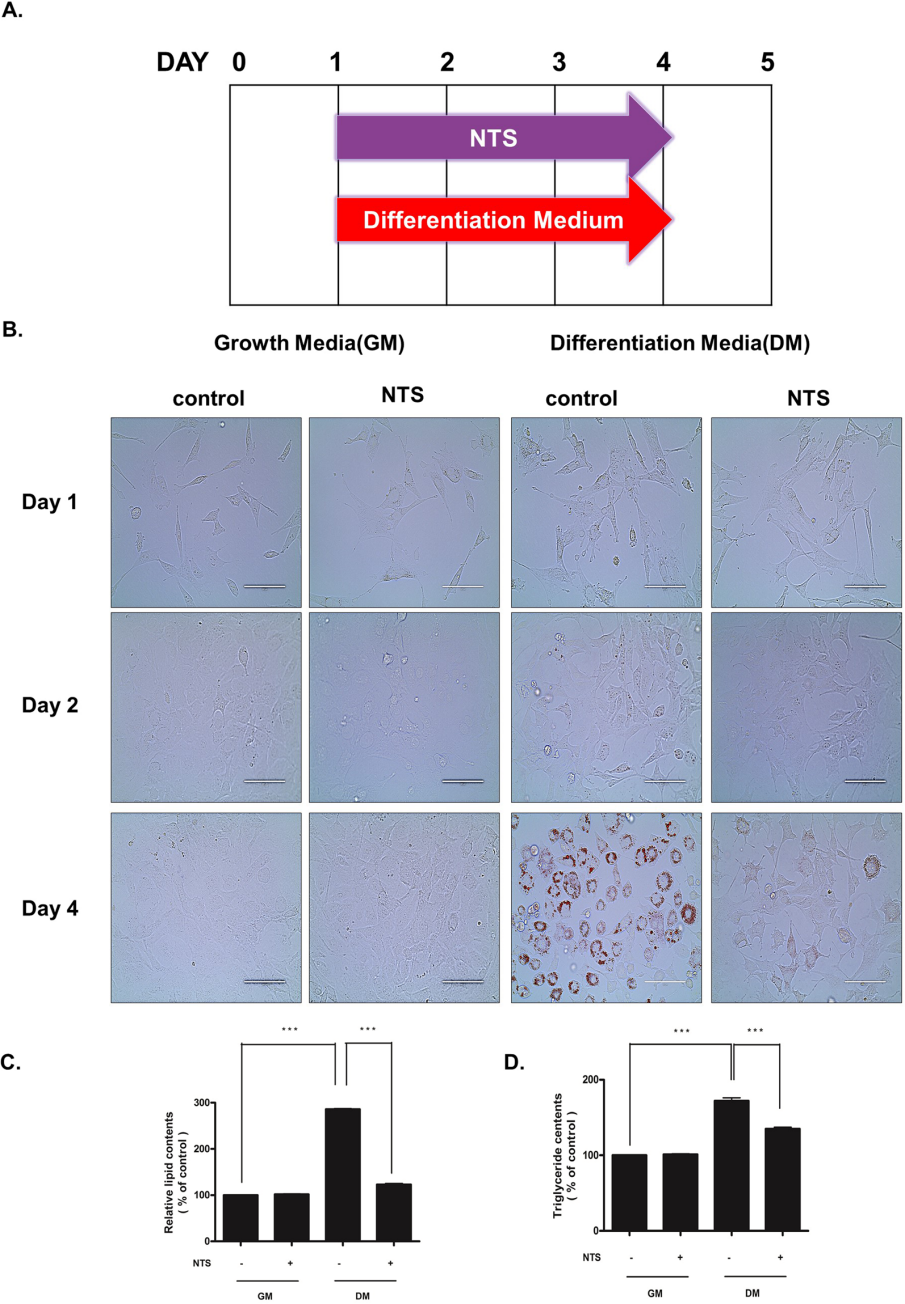
Inhibitory effect of NTS on adipogenic differentiation. (**A**) Schematic representation of the experimental design. (**B**–**D**) 3T3-L1 pre-adipocytes were treated with NTS for GM and DM and analyzed at the indicated time point after the start of adipocyte differentiation. (**A**) During four days of culture, adipocytes were stained with Oil Red O and then photographed for differentiation under a microscope. Scale bar = 100 μm. Inhibitory effect of NTS on (**B**) lipid content and (**C**) triglyceride deposition in differentiated 3T3-L1 cells. Results are presented as the mean ± SD of three independent experiments. Significant difference: ***p < 0.001 compared with the control group.

To determine the effect of NTS on adipogenic differentiation, we monitored intracellular lipid accumulation using Oil Red O staining, and 3T3-L1 pre-adipocytes cultured with differentiation medium differentiated into adipocytes. Results from Oil Red O staining and triglyceride contents assay showed that the staining intensity and triglyceride contents increased gradually in control cells (Supplementary Fig. 3). Adipogenic factors were also gradually induced (Supplementary Fig. 4). Interestingly, 3T3-L1 cells treated with NTS for four days showed significant inhibition of adipogenic differentiation (Fig. 3B). Furthermore, NTS significantly reduced the formation of oil droplets (to 67% ± 3%) compared with differentiated cells (100%, Fig. 3C).

We also examined triglycerides (TG) contents on day 4 in the differentiated cells. As shown in Fig. 3D, treatment with NTS significantly inhibited adipocyte differentiation (23% ± 2%) compared with NTS non-treated cells cultured with differentiation medium. There was no significant difference in adipocyte differentiation between the control and NTS treated cells cultured in the growth medium. These findings indicate that NTS treatment can dramatically inhibit adipogenic differentiation of 3T3-L1 cells.

### NTS inhibits adipogenic transcription factor and adipocyte specific gene expression in 3T3-L1 cells

**Figure 4:**
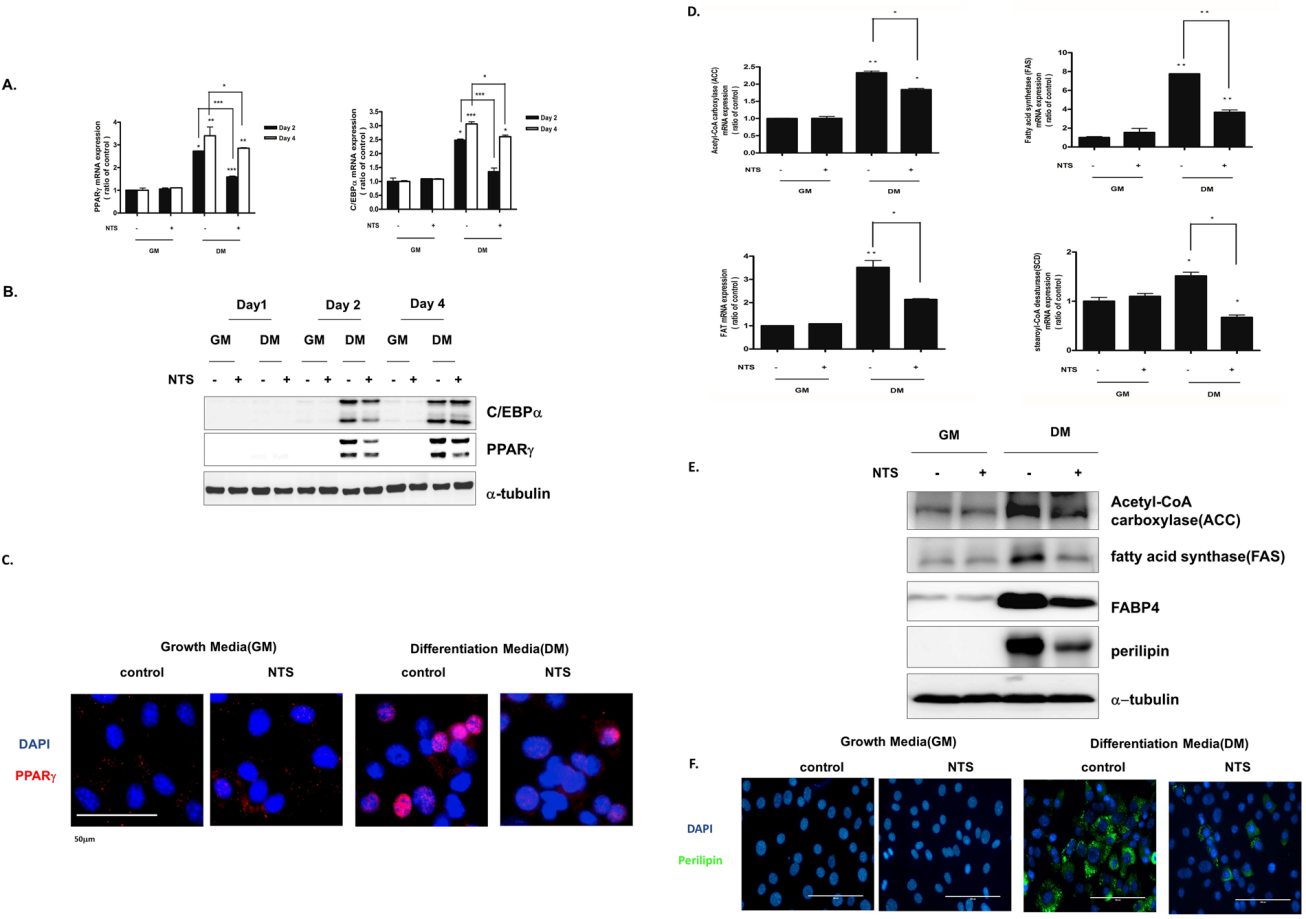
Inhibitory effect of NTS on key adipogenic transcription factors in 3T3-L1 pre-adipocytes. (**A**) Quantitative real-time PCR was used to quantify the effect of NTS on adipogenic transcription factors PPARγ and C/EBPα. Results are presented as mean ± SD of three independent experiments. Significant difference: ***p < 0.001; **p < 0.01; *p < 0.05 compared with the control group. (**B**) Western blot analysis showing the effect of NTS on PPARγ and C/EBP α levels. (**C**) Immunocytochemical assay for PPARγ (adipogenic transcription factor, Red) and DAPI (nucleus stain, Blue). Scale bar = 100 μm. (**D**) On day 4, adipocyte specific markers (fatty acid synthase, ACC-acetyl-CoA carboxylase, FAT, and SCD-1-stearoyl-CoA desaturase) were analyzed by real-time PCR using mRNA isolated from NTS-treated 3T3-L1 pre-adipocytes. (**E**) Proteins were extracted and expression levels of FAS, ACC, FABP4, and perilipin were determined by Western blot. Each Western-blotting band represents three experiments performed in triplicate. (**F**) Immunocytochemical assay for perilipin (lipid droplet marker, green) and DAPI (nucleus stain, blue). Scale bar = 100 μm.

In adipogenic differentiation, adipogenic transcription factors PPARγ and C/EBPα are well-known to regulate pre-adipocyte differentiation into adiopcytes^22^. In this study, we evaluated the effect of NTS on adipogenic differentiation-associated gene expression by qPCR. In the absence of NTS treatment, mRNA levels of PPARγ and C/EBPα increased when adipocytes were cultured in adipogenic differentiation medium (Supplementary Fig. 4A). Interestingly, NTS treatment significantly reduced mRNA levels of PPARγ and C/EBPα on day 2 and day 4. However, this reduction was not detected in growth medium the groups (Fig. 4A). These results suggest that NTS treatment could inhibit adipogenesis at the transcriptional level.

To understand the mechanism by which NTS treatment inhibited adipogenic differentiation at the protein level, we performed Western blot analysis. Consistent with gene expression patterns, adipogenesis-associated proteins (PPARγ, C/EBPα, perilipin, acetyl CoA carboxylase, fatty acid synthesis, and FABP4) were induced gradually when we cultured the cells in adipogenic medium (Supplementary Fig. 4B). NTS treatment inhibited PPARγ and C/EBPα expression on day 2. However, NTS did not inhibit C/EBPα expression on day 4 (Fig. 4B). Additionally, we performed immunocytochemistry analysis, and the results showed that PPARγ was localized in the nucleus of differentiated 3T3-L1 adipocytes, not pre-adipocytes (Fig. 4C).

Next, we determined the effect of NTS on the expression of adipocyte-associated genes such as ACC, FAS, SCD1, and FAT with real-time PCR. As shown in Fig. 4D, mRNA levels of adipogenic-associated genes including ACC, FAS, and FAT SCD1 were significantly decreased in the NTS-treated group. Consistent with mRNA expression patterns, ACC and FAS protein levels were also significantly reduced by NTS treatment.

It has been previously reported that perilipins and FABP4 have a regulatory role during lipid droplet formation of adipogenesis^23–26^. As shown in Fig. 4E, immunofluorescence microscopy (Fig. 4F). Perilipin expression was localized in the lipid droplets in differentiated 3T3-L1 adipocytes but not in the undifferentiated cells (Supplementary Fig. 4D). Similar to the results shown in Fig. 4E, we confirmed that perilipin lipid droplet staining was significantly reduced in the NTS-treated group (Fig. 4F) compared with in the non-treated group. These findings indicate that NTS treatment can dramatically inhibit adipogenesis-associated gene expression and adipogenic characteristics.

### NTS inhibits late-stage adipocyte differentiation in 3T3-L1 cells

**Figure 5:**
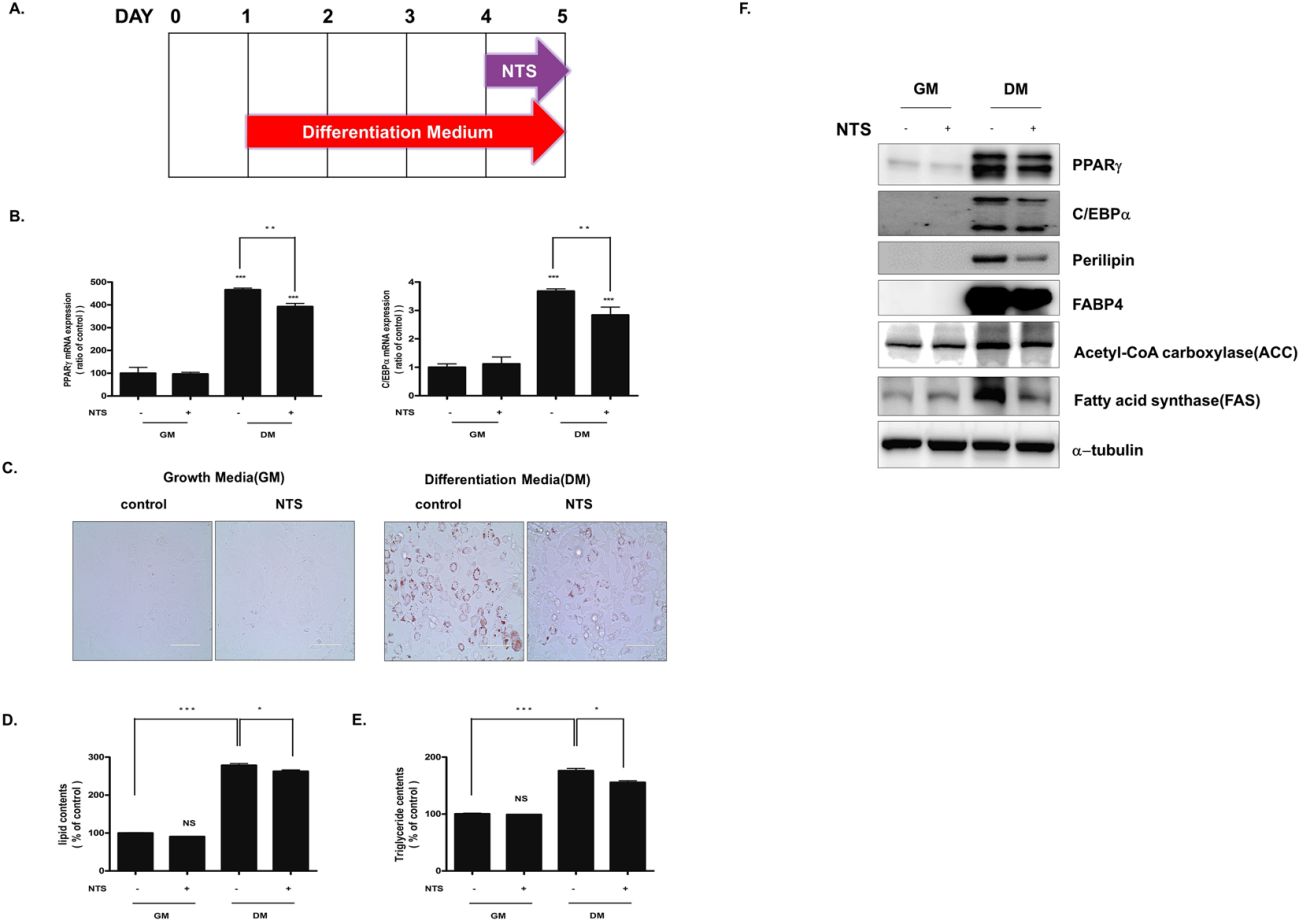
Inhibitory effect of NTS during the late stage of adipogenic differentiation. (**A**) Schematic representation of the experimental design. (**B**–**E**) 3T3-L1 preadipocyte cells were cultured in GM and DM and treated with NTS on day 4. (**B**) Quantitative real-time PCR was used to quantify the effect of NTS on adipogenic transcription factors PPARγ and C/EBPα. (**C**) Oil Red O staining. Scale bar = 100 μm. (**D**) Lipid content. (**E**) Triglyceride deposition. Results are presented as mean ± SD of three independent experiments. Significant difference: ***p < 0.001, *p < 0.05 compared with the control group. (**F**) Western blot assay. Each Western-blotting band is a representative of three experiments performed in triplicate.

To determine whether NTS had an inhibitory effect on late-stage adipocyte differentiation, we added NTS to differentiating cells on day 4. We then examined lipid accumulation and adipogenesis-associated gene expression on day 5 (Fig. 5). In addition, we also evaluated mRNA levels of PPARγ and C/EBPα in NTS-treated cells. As shown in Fig. 5B, although NTS treatment significantly reduced mRNA PPARγ and C/EBPα expression, their expressions were less inhibited in the late stage than the early stage of differentiation.

In addition, we examined the accumulation of intracellular lipids with Oil Red O staining. As shown in Fig. 5C, NTS treatment significantly inhibited 3T3-L1 cell differentiation compared with the control cells. Lipid accumulation in differentiated adipocytes was also significantly reduced in NTS-treated cells (Fig. 5D).

To confirm the extent of suppression of adipocyte differentiation by NTS, we performed TG content assay and Western blot analysis. As shown in Fig. 5E, TG content decreased to 89% by NTS. Moreover, protein levels of the adipocyte-specific markers PPARγ, C/EBPα, ACC, FAS perilipin, and FABP4 decreased significantly in NTS-treated cells (Fig. 5F). These results suggest that NTS is a potent inhibitor during both early-stage and late-stage adipogenesis in 3T3-L1 cell differentiation.

### NTS inhibits ER stress and UPR activation during adipocyte differentiation in 3T3-L1 cells

To identify the underlying mechanism of the effects of NTS, we assessed whether NTS regulated ER stress and UPR activation. Previous studies have described that ER stress is a requirement for 3T3-L1 pre-adipocyte cells differentiating into adipocytes^6,27^. Therefore, we examined several ER stress markers in this study. As shown in Supplementary Fig. 5, expression levels of UPR and ER stress markers including GRP78, p-IRE1α, p-PERK, p-eIF2α, and CHOP were up-regulated in adipocytes at the beginning of differentiation, although XBP1 expression did not increase. Recently, it was reported that CHOP is induced through eIF2α phosphorylation. It suppressed adipogenesis by interfering with other C/EBP family members^28^. Thus, we examined CHOP expression during adipogenesis using immunocytochemistry; interestingly, CHOP was localized in the nucleus of differentiated 3T3-L1 adipocytes (Supplementary Fig. 5B). These results suggest that ER stress and UPR activation are among the essential components of adipogenesis.

**Figure 6:**
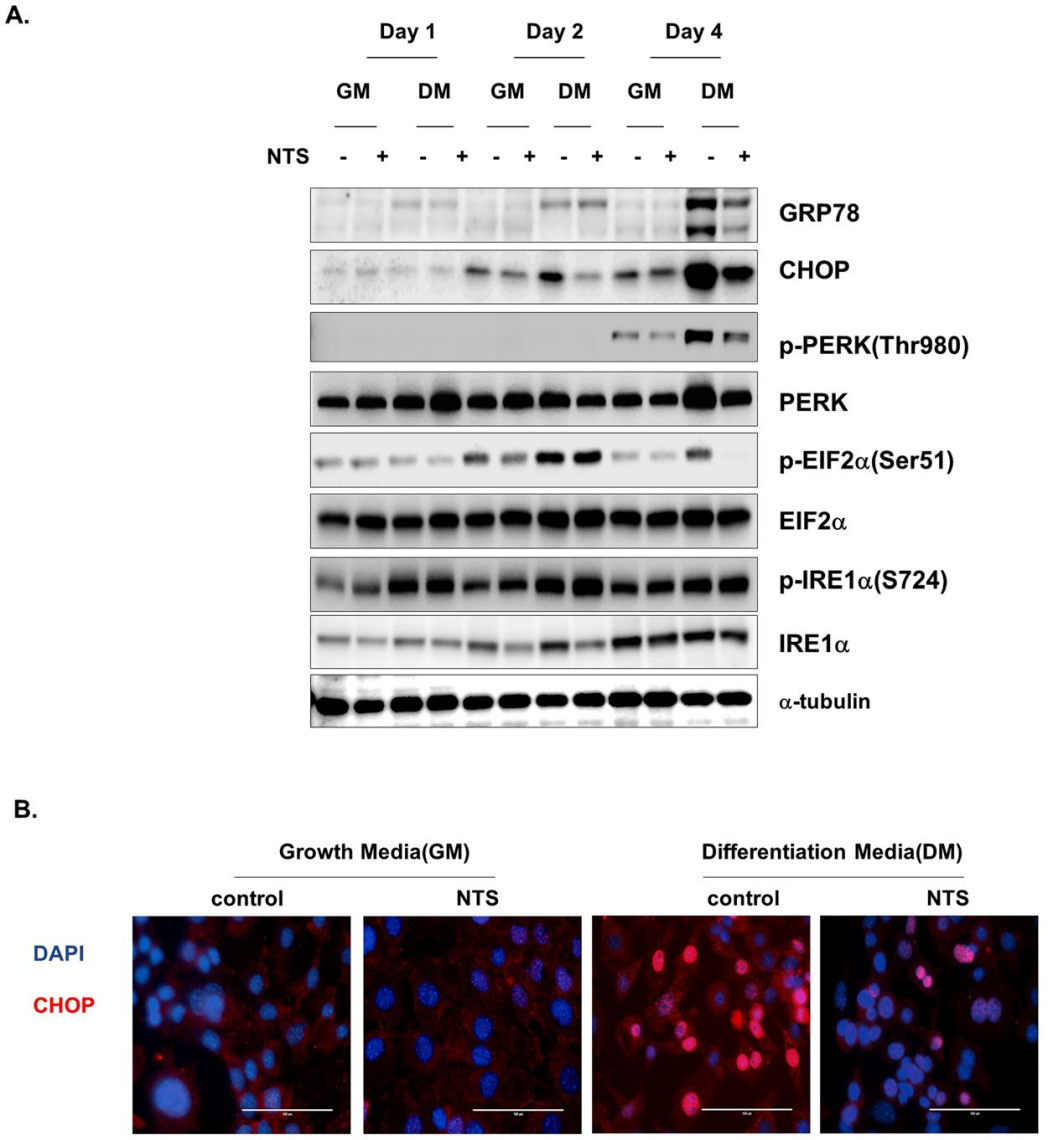
NTS inhibits ER stress and UPR activation as it inhibits 3T3-L1 adipocyte differentiation. (**A**) Protein expression profiles for ER stress-related protein and UPR related protein. Cell lysates were harvested at the indicated time points after the start of adipocyte differentiation and analyzed by Western analysis. Each Western-blotting band represents three experiments performed in triplicate. (**B**) Immunocytochemical assay for CHOP (ER stress marker, red) and DAPI (nucleus stain, blue). Scale bar = 100 μm.

Next, we examined whether NTP inhibited ER stress and UPR activation during adipogenic differentiation. Treating 3T3-L1 cells with NTS on day 4 dramatically inhibited GRP78, CHOP, p-PERK, and p-eIF2α in 3T3-L1 cells compared with in non-NTS-treated control cells (Fig. 6A), although another UPR molecule, p-IRE1α, was not inhibited. Additionally, we analyzed CHOP translocation to the nucleus using immunocytochemistry; as expected, NTP effectively inhibited the nuclear translocation (Fig. 6B). These data showed that NTS can strongly suppress adipogenic differentiation of pre-adipocytes by inhibiting ER stress and UPR activation.

### Effects of NTS on the decreases adipose tissue mass and adipocyte size in C57B/6 Mice Fed High-fat Diets

**Figure 7:**
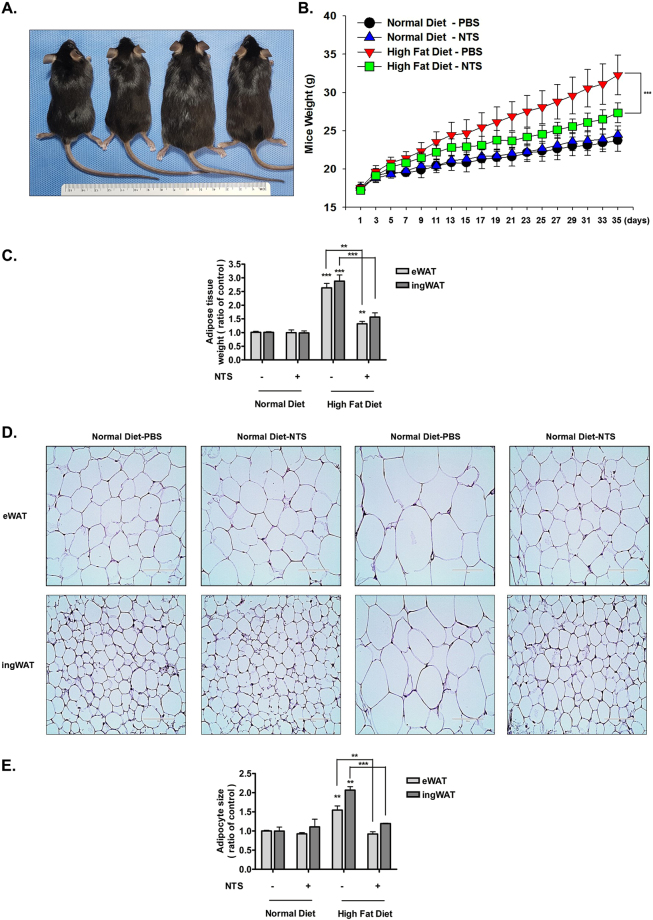
Effect of NTS on body weight and adipogenesis in mice fed with a high-fat diet. C57BL/6 mice were fed with a high-fat diet or normal diet for 5 weeks. Each group was divided into two groups, one group was injected with PBS, and the other group was injected with NTS. (**A**) The photograph was taken 5 weeks after NTS or PBS treatment. From the left, normal diet/ PBS, normal diet/NTS, high-fat diet/PBS, and high-fat diet/NTS. (**B**) Effect of NTS on weight gain. From day 21, high-fat diet-fed mice injected with NTS had significantly lower body weights compared with PBS-injected mice. Significant difference: ***p < 0.001 compared with the control group. N = 10. (**C**) Effect of NTS on adipose tissue weight. eWAT and ingWAT were isolated from all groups. The high-fat diet-fed mice injected with NTS had significantly lower mean adipose tissue weight in comparison to the mice injected with PBS. Significant difference: ***p < 0.001, **p < 0.01 compared with the control group. N = 10 (**D**) H & E staining of adipose tissue. Representative pictures from the groups are shown. Scale bar = 100 μm. (**E**) Adipocyte size of high-fat diet-fed mice is reduced by NTS treatment. High-fat diet-fed mice injected with NTS had a significant reduction in average cell size compared with the high-fat diet-fed mice injected with PBS. Significant difference: ***p < 0.001, **p < 0.01 compared with the control group. N = 10.

Given our *in vitro* experiment results, we sought to determine whether NTS decreased body weight by inhibiting the adipose tissue. Next, we confirmed the inhibitory effect of NTS on adipocytes by using a mouse model of diet-induced obesity. Although their food intake did not differ (Supplementary Fig. 7), the NTS-injected mice displayed decreased body fat and less lean body mass compared to PBS-injected mice (Fig. 7A,B), which was thought to be due to an increase in adipose tissue mass. Thus, eWAT and ingWAT were isolated from all groups of mice. The weights of eWAT and ingWAT were significantly reduced in NTS-injected mice, compared to the untreated group (Fig. 7C).

Next, we examined the effect of NTS on adipocyte differentiation and cell size. As shown in Fig. 7D, the number of adipocytes was significantly lower in the NTS-injected groups than in the non-treated group. In addition, the mean cell size in the NTS-injected group was significantly smaller than those of the other groups, as demonstrated graphically in Fig. 7E. Furthermore, histological analysis revealed the presence of a fat liver in all mice, and significantly decreased triglyceride accumulation was found in the NTS-injected mice (data not shown).

## Discussion

Several studies have suggested that obesity is a risk factor for metabolic diseases such as diabetes, hypertension, and cardiovascular disease^1,2^. Thus, many therapeutic strategies for obesity have been proposed; however, no effective drug treatment is currently available.

Results of this study demonstrated that NTS inhibited the expression of adipogenic transcription factor and adipocyte-associated genes, lipid accumulation, and triglyceride contents in pre-adipocytes, suggesting that NTS might be useful for inhibiting obesity.

Recently, the development of innovative new type of plasma devices generating NTP has improved their potential applications especially in biology and medicine. Our previous studies showed that NTP could be used as a novel treatment for a variety of applications such as cancer cell death, wound healing, and muscle regeneration^18–20,28^. However, due to the permeability into the epidermis, direct NTP treatment has limited usefulness inside the body. Therefore, in the previous report, we developed NTS, a liquid form of NTP, to confirm that the effects of NTS is not different from that NTP effects. Therefore, we developed NTS, a liquid form of NTP and confirmed that the effect of NTS is same to that of NTP^21^. Thus, we applied NTS to inhibit adipogenesis, and showed that NTS has inhibitory effect on adipogenesis without toxicity or apoptotic effect on 3T3-L1 (Supplementary Fig. 1).

Obesity is known to be caused by overgrowth of adipocyte mass due to increased numbers and/or sizes of adipocytes differentiated from pre-adipocytes^1–4^. Our results showed that NTS did not inhibit adipocyte proliferation or viability, but it inhibited lipid accumulation and adipogenic transcription factors such as PPARγ and C/EBPα (Fig. 3).

Many studies have shown that UPR and ER stress are activated during adipocyte differentiaiton^27,29^. For example, phosphorylated eIF2 and CHOP is an integrated stress response; they regulate PPARγ and C/EBPα. Consistent with these findings, our results demonstrated that ER stress (GRP78, CHOP, and p-eIF2α) and UPR markers (p-IRE1α and p-PERK) were induced by adipogenic differentiation medium on day 4 (supplementary Fig. 5)^30,31^. This result is reasonable given that CHOP is a known regulator of adopogenesis^29^ and up-regulation of CHOP following treatment with ER stress inhibitor can block this process^32^. In addition, a recent study showed that increased CHOP levels can play other metabolic roles within cells by binding to C/EBPα promoter regions involved in lipogenic gene inhibition in the liver^33^. NTS treatment effectively suppressed ER stress and UPR response during adipogenic differentiation. It also inhibited nucleus translocation and CHOP protein expression on day 4 in the late period of 3T3-L1 differentiation (Fig. 6).

In contrast, recent report showed that overexpression of IRE1a, an UPR signaling, did not alter adipogenic differentiation^27^. These results indicate that although ER stress is important, various factors are associated with adipogenic differentiation. Therefore, it seems that ER stress and UPR activation play a very important role in lipogenesis but that cross-talk between adipocytes and other signal transductions might be involved heavily in the lipogenesis.

Given that NTS inhibits ER stress and adipogeneic differentiation in a cell culture system, we expected NTS to act similarly *in vivo*, leading to the inhibition of body fat production. Thus, NTS was injected into the abdomen through intraperitoneal injection for easy accession of the plasma, as much as possible, to the adipose tissue. Consistent with our *in vitro* findings, NTS-injected mice had decreased weight and adipose size in their eWAT and ingWAT (Fig. 7). NTS- injected mice had significantly lower body fat and body fat weight on average. *In vitro* experimental results showed that NTS treatment at various stages of adipogenesis affected lipid storage and adipogenesis. Thus, NTS treatment in mice is likely not only affecting adipocyte differentiation but also playing a role in the maturation stage.

In this study, we demonstrated that blocking ER stress/UPR signaling by plasma treatment can prevent adipocyte differentiation in 3T3-L1 cells and in the adipose tissue of high-fat diet-fed C57BL/6 mice. However, further studies on the genetic predisposition to lipid production and adipogenesis should be conducted. Although more studies might be necessary to determine more detailed mechanisms of the plasma involved in inhibiting adipocyte differentiation, our results indicated that NTS might be used as a potential inhibitor of adipogenic differentiation by inhibiting ER stress and UPR activation.

## Electronic supplementary material

Supplementary Information
