# Supplementary material for: Nonthermal plasma treated solution inhibits adipocyte differentiation and lipogenesis in 3T3-L1 preadipocytes via ER stress signal suppression
Source: Sci Rep. 2018 Feb 2;8:2277. doi: 10.1038/s41598-018-20768-5 (PMC5797163; doi:10.1038/s41598-018-20768-5)
Supplement: Supplementary file 1 — Supplementary Information [file 41598_2018_20768_MOESM1_ESM.pdf]

Non-thermal Plasma treated solution inhibits adipocyte differentiation and lipogenesis in 3T3-L1 preadipocytes via ER stress signal suppression

Sung Un Kang[1#], Haeng Jun Kim[1,2#], Dae Ho Kim[1,2], Chang Hak Han[1,2], Yun Sang Lee[1], Chul-Ho Kim[*1, 2]

Supplementary Figure 1.

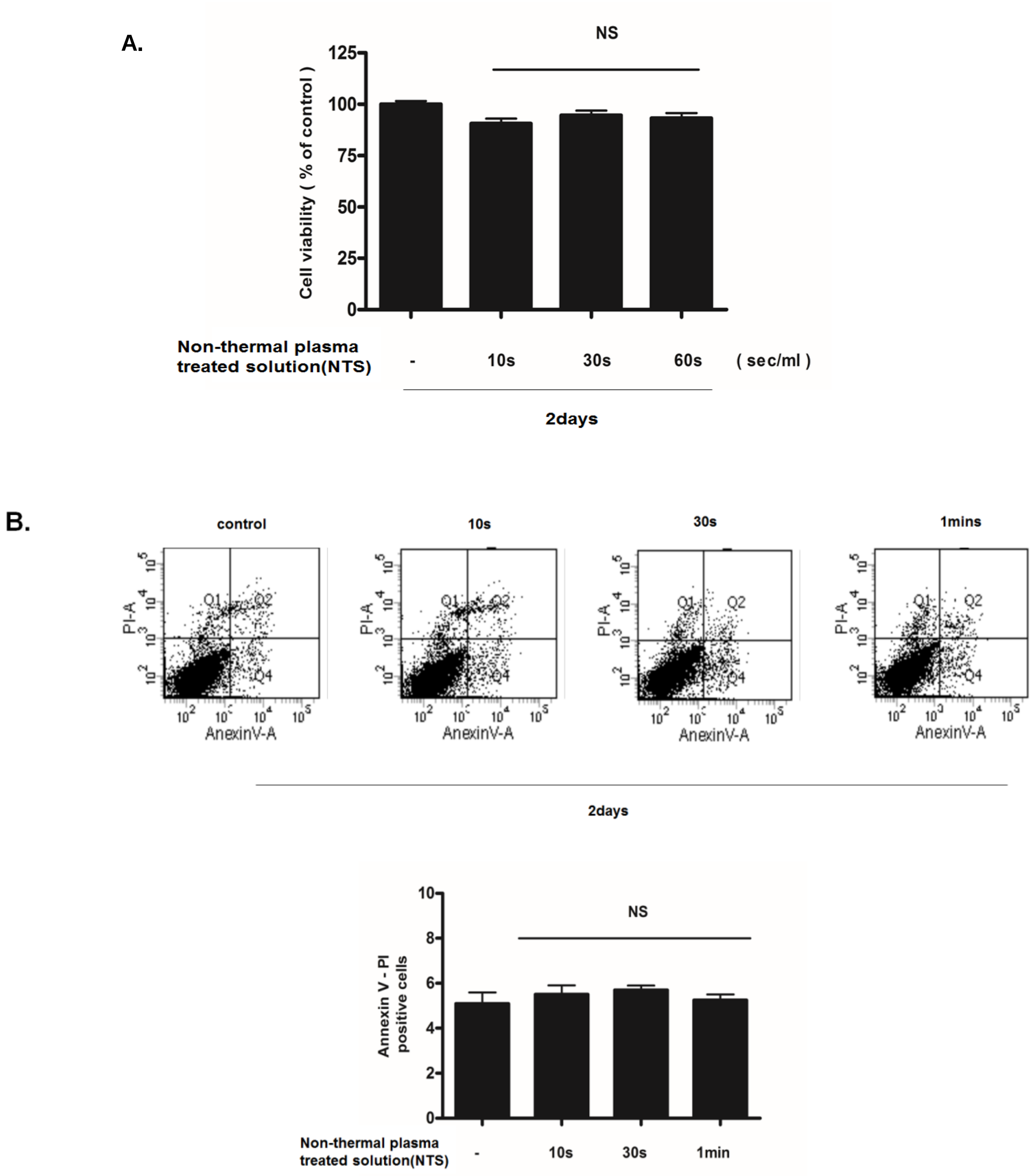

C.

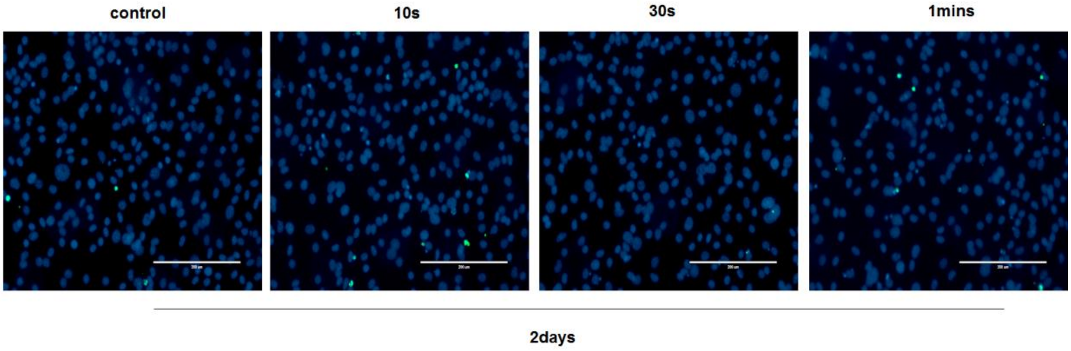

Supplementary Figure 1. Assessment of cytotoxicity and cell death effect of NTS (4 kV 1 ml / 1 min) treatment on 3T3-L1 preadipocytes. (A) Cell proliferation and cytotoxicity were determined by MTT assay. Bar graph represents mean ± standard deviation of three-independent experiments. NS, not significant; **, P < 0.01. (B) Annexin V/PI assay and quantification of preadipocyte. Bar graph: mean ± S.D. of three independent experiments. NS, not significant. (C) TUNEL assay. Scale bars = 100 μm.

Supplementary Figure 2.

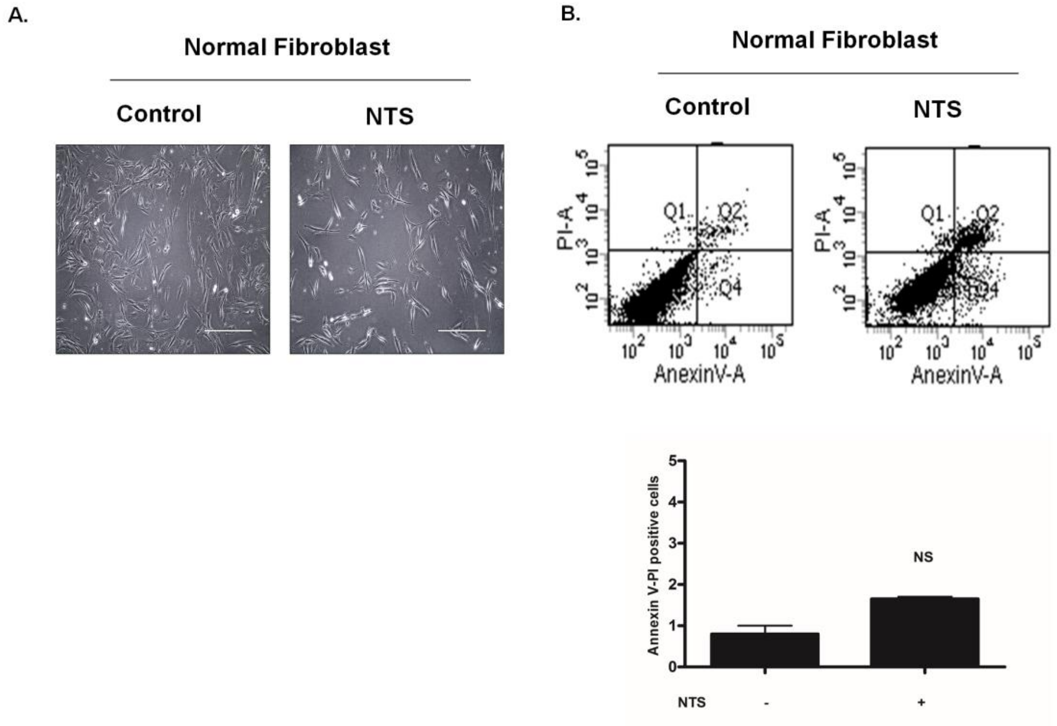

Supplementary Figure 2. Cytotoxic and cell death effect of NTP treatment on normal fibroblast. (A) Phase contrast microscope. Scale bar = 400 mm. (B) Annexin V/PI assay and quantification of normal fibroblast. Bar graph: mean ± S.D. of three independent experiments. NS, not significant.

Supplementary Figure 3

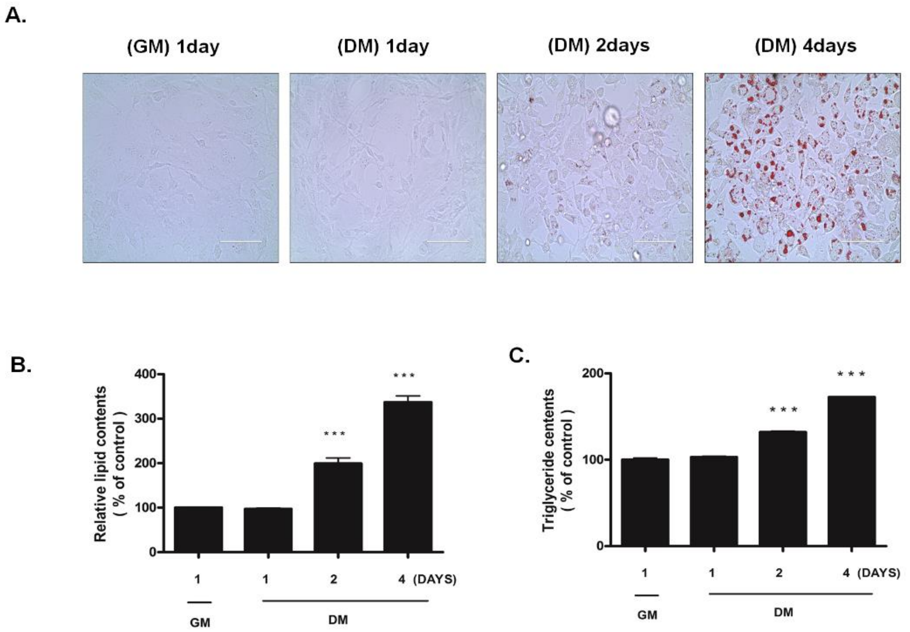

Supplementary Figure 3. Assessment of adipogenic differentiated effect on adipogenic differentiation media in 3T3-L1 preadipocytes.

(A) Differentiated adipocyte cells were stained with Oil Red O and then photographed using a microscope. Scale bar = 100 µm. (B) Inhibitory effect of NTS on lipid content. (C) Inhibitory effect of NTS triglyceride deposition in differentiated 3T3-L1 cells. Results are presented as mean ± SD of three independent experiments. Significant difference: ***, $p < 0.001$ as compared to the control group.

Supplementary Figure 4

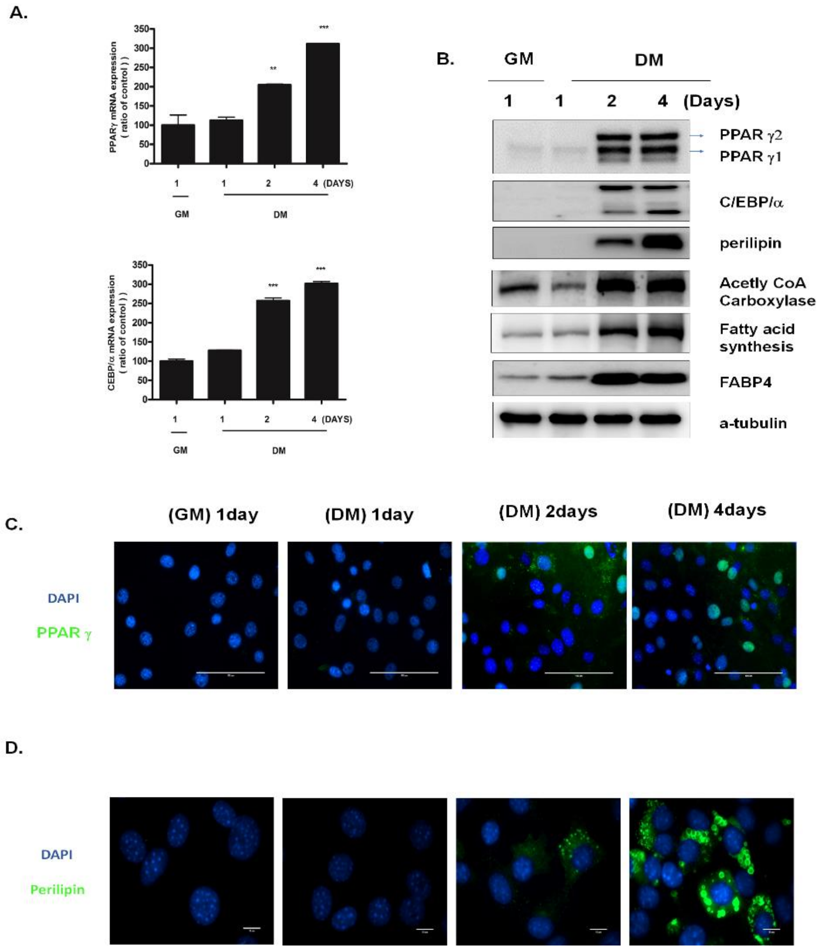

Supplementary Figure 4. Assessment of adipogenic transcription factor and adipocyte related protein expression on adipogenic differentiation media in 3T3-L1 preadipocytes.

(A) Quantitative Real-Time PCR was used to quantify the effect of NTS on adipogenic transcription factors PPARγ and C/EBPα. Results are presented as mean ± SD of three independent experiments. Significant difference: ***, $p < 0.001$; **, $p < 0.01$; *, $p < 0.05$ as compared to the control group. (B) Western blot analysis showing the effect of NTS on PPARγ and C/EBPα protein levels. (C, D) Immunocytochemical assay for PPARγ (adipogenic transcription factor, green), Perilipin (lipid droplet marker, green), and DAPI (nucleus stain, Blue). Scale bar = 100 μm.

Supplementary Figure 5

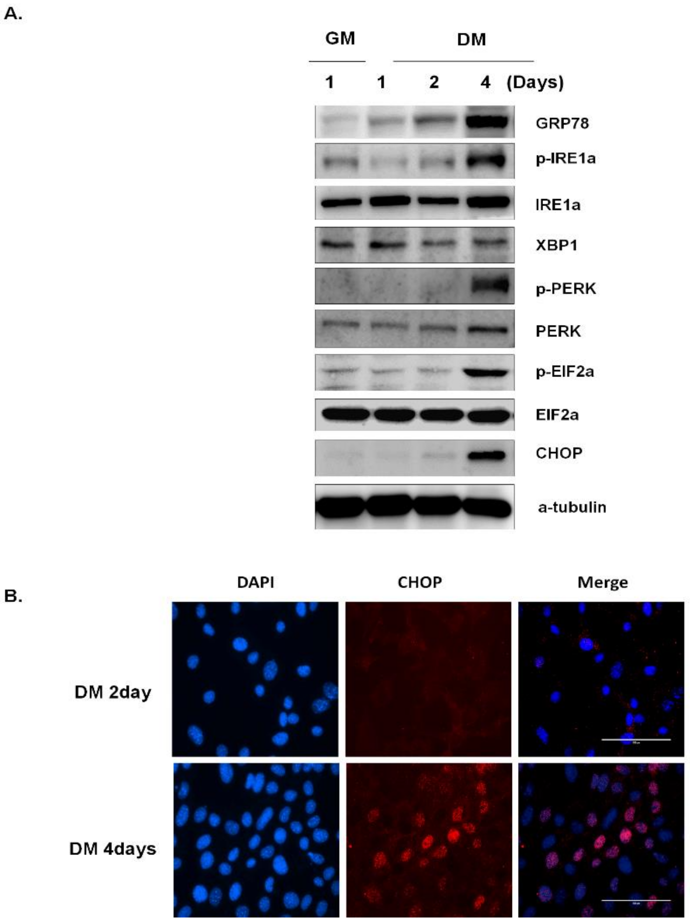

Supplementary Figure 5. Assessment of ER stress and UPR expression on adipogenic differentiation media in 3T3-L1 preadipocytes. (A) Protein expression profiles for ER stress related protein and UPR-related protein. Cell lysates were harvested at indicated time points after the start of adipocyte differentiation and analyzed by western blotting analysis. Each Western-blotting band is a representative of three experiments performed in triplicates. (B) Immunocytochemical assay for CHOP (ER stress marker, Red) and DAPI (nucleus stain, Blue) on day 2 day and day 4. Scale bar = 100 μm.

Supplementary Figure 6

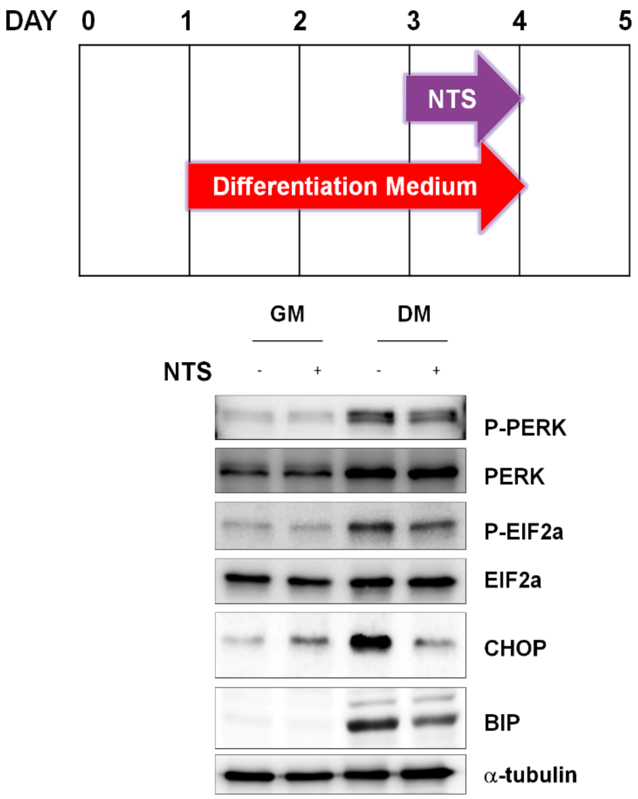

Supplementary Figure 6. Inhibitory effect of NTS on ER stress related protein after adipogenic differentiation.

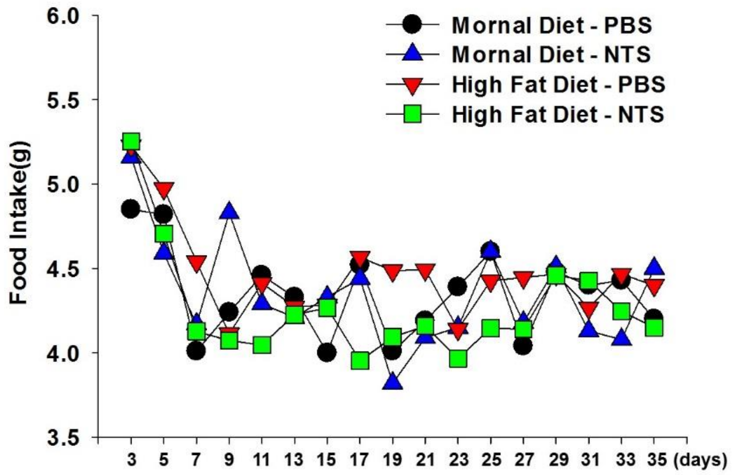

Supplementary Figure 7. Food intake measurements. No significant difference in food intake was noted between the four groups.
